# A Novel Interaction between TFII-I and Mdm2 with a Negative Effect on TFII-I Transcriptional Activity

**DOI:** 10.1371/journal.pone.0144753

**Published:** 2015-12-11

**Authors:** Kateřina Cetkovská, Hana Šustová, Pavlína Kosztyu, Stjepan Uldrijan

**Affiliations:** 1 International Clinical Research Center—Center of Biomolecular and Cellular Engineering, St. Anne's University Hospital, Brno, Czech Republic; 2 Department of Biology, Faculty of Medicine, Masaryk University, Brno, Czech Republic; Georgia Regents University, UNITED STATES

## Abstract

Williams-Beuren syndrome-associated transcription factor TFII-I plays a critical regulatory role in bone and neural tissue development and in immunity, in part by regulating cell proliferation in response to mitogens. Mdm2, a cellular oncogene responsible for the loss of p53 tumor suppressor activity in a significant proportion of human cancers, was identified in this study as a new binding partner for TFII-I and a negative regulator of TFII-I-mediated transcription. These findings suggest a new p53-independent mechanism by which increased Mdm2 levels found in human tumors could influence cancer cells. In addition to that, we present data indicating that TFII-I is an important cellular regulator of transcription from the immediate-early promoter of human cytomegalovirus, a promoter sequence frequently used in mammalian expression vectors, including vectors for gene therapy. Our observation that Mdm2 over-expression can decrease the ability of TFII-I to activate the CMV promoter might have implications for the efficiency of experimental gene therapy based on CMV promoter–derived vectors in cancers with Mdm2 gene amplification.

## Introduction

A tight control of gene expression is critically important for proper regulation of cell growth, proliferation, survival and differentiation during development and for the maintenance of tissue homeostasis in a multicellular organism. This is achieved by a large number of transcription factors involved in the spatial and temporal control of gene activity in response to intra- and extracellular signals.

Williams-Beuren syndrome, a complex multisystemic genetic disorder characterized by a unique cognitive profile and craniofacial defects, results from a small deletion at the chromosomal location 7q11.23 that encompasses, among other genes, *GTF2I*, *GTF2IRD1* and *GTF2IRD2*, encoding three members of TFII-I family of transcription factors [1]. In mice, the homozygous loss of either *Gtf2ird1* or *Gtf2i* gene function results in multiple phenotypic manifestations, including embryonic lethality, brain hemorrhage, craniofacial malformations and defects in vasculature and neural tube development [2]. Other studies showed that TFII-I plays an important role in regulating genes that are essential in osteogenesis [3].

TFII-I family members contain several repeats of a unique folded structural motif, so-called I-fold, within a region similar to the helix-loop-helix (HLH) motif. Despite not being a classical HLH protein, TFII-I is able to functionally behave as a HLH protein in certain ways, including interactions with USF protein and E-box sequence motifs in DNA. In addition to binding E-box, TFII-I can recognize and bind the Initiator element (*Inr*) and *Inr*-like DNA sequences [4]. Alternative splicing produces several TFII-I variants in human cells but their biological role remains largely uncharacterized. Experiments in mouse cells suggest that some TFII-I isoforms can activate target gene expression, while other isoforms might be preferentially involved in transcriptional repression, with both groups competing for the same specific binding sites [5]. The repressive function of TFII-I is mediated by interactions with transcription co-repressors such as the polycomb repressor complex and histone deacetylases HDAC1 and HDAC3 [6–9].

TFII-I participates in the regulation of a number of diverse cellular processes, including cell proliferation, immune signaling in B- and T-cells or the endoplasmic reticulum stress response [4]. TFII-I is involved in the transcriptional activation of the *c-fos* and *cyclin D1* genes in response to extracellular mitogenic stimuli such as the epidermal growth factor (EGF) and the platelet-derived growth factor (PDGF) [10–12]. Moreover, TFII-I can physically interact with the extracellular signal-regulated kinase (ERK) and mediate its nuclear translocation in response to mitogens [13]. There is also a report linking TFII-I regulation to DNA damage response by showing that TFII-I is ubiquitinated and proteasomally degraded in response to genotoxic stress in a manner dependent on the function of tumor suppressors ATM (ataxia telangiectasia mutated) and p53 [14].

Tumor suppressor p53 regulates cellular responses to various types of stress stimuli, e.g. hypoxia or DNA damage, and p53 activation normally leads to cell cycle arrest or apoptosis of damaged cells [15]. Mouse double minute 2 (Mdm2), the product of a p53 target gene, serves as an E3 ubiquitin ligase for p53 and a critical negative regulator of p53 protein levels and transcriptional activity in normal untransformed cells. However, Mdm2 over-expression is responsible for the loss of p53 function in a significant proportion of human cancers [16]. In addition to its role in p53 regulation, there is a growing body of evidence for p53-independent regulation of cell signaling and proliferation by Mdm2 and a related oncogene Mdm4 [17–21]

Here we present results suggesting that Mdm2 can interact with TFII-I protein, encoded by the human *GTF2i* gene, and this can have negative effects on TFII-I-dependent transcription in human cells. We also present data indicating that TFII-I can regulate, in addition to its normal cellular targets, the transcriptional activity of the frequently used immediate-early promoter of human cytomegalovirus (CMV promoter). The interaction between Mdm2 and TFII-I might therefore have negative implications for CMV promoter-based gene therapy in cancers over-expressing Mdm2.

## Materials and Methods

### Cell culture

Human U2OS, H1299 and HEK293T cell lines (obtained from the European Collection of Animal Cell Cultures, ECACC, Salisbury, United Kingdom) were cultivated at 37°C and 5% CO_2_ in a high-humidity atmosphere in Dulbecco’s modified Eagle’s medium (DMEM, Sigma-Aldrich) supplemented with 10% fetal bovine serum (FCS), 2 mM glutamine, 100 U/ml penicillin and 100 μg/ml streptomycin sulfate.

### Plasmid constructs

Plasmids pEBG/TFII-I coding for GST-tagged wild type TFII-I (GST-TFII-I) and GST-tagged TFII-I mutant lacking the NLS signal (GST-TFII-IΔNLS) [22] and the c-*fos*-promoter luciferase reporter plasmid (pSVOAΔ5’ *c-fos*-Luc) were kindly provided by Dr. Ananda L. Roy (Tufts University School of Medicine, Boston, U.S.A.). The pEBG plasmid backbone used in the GST-TFII-I and GST-TFII-IΔNLS constructs allows for mammalian expression of GST fusion proteins under the control of the human elongation factor 1-α promoter. For the expression of untagged TFII-I, the GST tag was removed by cloning the *ClaI* fragment from pEBG/TFII-I into the *BstBI* site of pcDNA4/myc-His (Life Technologies). Plasmids coding for luciferase under the control of the full length CMV promoter or its deletion mutants (pGL3 CMVdel1—CMVdel6) [23] were kindly provided by Dr. Yu-Chan Chao (Institute of Molecular Biology, Taipei, Taiwan). In our study we did not use the CMVdel2 construct of the series as it failed to induce significant levels of luciferase activity in our cell models. Plasmids pcDNA3-Flag-Arf and pCHDM1A, coding for FLAG-tagged human ARF protein and wild-type human Mdm2, respectively, were kindly provided by Prof. Karen H. Vousden (Beatson Institute for Cancer Research, Glasgow, UK). Plasmid pcDNA3-Flag-USP48 was kindly provided by Prof. George Mosialos (Aristotle University of Thessaloniki, Greece). Plasmids pcDNA3.1(-)/myc-His/LacZ and pcDNA4/LacZ, coding for β-galactosidase under the control of CMV promoter, were obtained from Life Technologies. Plasmids pCEP4Tat and pHIVlacZ for β-galactosidase expression under the control of the U3 region and a part of the R region of the HIV-1 3′ LTR have been described previously [24,25]. Plasmids coding for enhanced GFP protein (pEGFP-N1 and pEGFP-C2) were obtained from Clontech. Plasmid pEGFP-Sp1 encoding GFP-tagged full length Sp1 has been described previously [26]. In all transfections, the total amount of DNA was kept constant using empty plasmids pcDNA3 (Invitrogen) or pEBG (kindly provided by Dr. Ananda L. Roy).

### Down-regulation of TFII-I and Mdm2 expression

Mixtures of small interfering RNAs (siRNAs) targeting TFII-I and non-targeting controls were purchased from Santa Cruz Biotechnology. The sequence of siRNAs targeting Mdm2 (5´-GCC ACA AAU CUG AUA GUA -3´) was published previously [21]. Hiperfect transfection reagent (QIAGEN) was used for siRNA transfection into human cell lines according to the manufacturer’s protocol. SiRNAs were transfected 24 hours prior to DNA transfection and cells were cultivated for additional 48 h.

### Luciferase assays

U2OS cells on 12-well plates were transfected with CMVdel1–6 series luciferase plasmid constructs or pSVOAΔ5’ *c-fos*-Luc construct, TFII-I and Mdm2 plasmids using TurboFect reagent (Thermo Scientific) according to the manufacturer’s instructions. Luciferase activity in cells 24 hours post-transfection was measured using the Luciferase Assay System Kit (Promega) according to the manufacturer’s instructions. For the analysis of the effect of TFII-I or Mdm2 knockdown on CMV promoter-driven luciferase expression, siRNAs were transfected 24 h prior to DNA transfection. The absolute values of luciferase activity were recalculated in relation to total protein concentration in the sample. Luciferase assays were performed as three independent experiments. Results are presented as means +/- standard deviations and two-tailed Student’s t test was used to determine P values (GraphPad InStat3 software).

### β-galactosidase assays

U2OS or H1299 cells on 12-well plates were transfected using the TurboFect reagent, collected 24 h post-transfection into PBS using cell scraper and centrifuged 5 min at 1500 rpm. Cell pellet was resuspended in 400 μl of 0.25 M Tris (pH 7.5). Cells were lysed by sonication and lysates cleared by centrifugation (15000 rpm/20 min/4°C). The amount of cell lysate containing 25 μg of total protein was incubated (30 min at 37°C) with 3 μl of 100x Mg (0.1 M MgCl_2_, 4.5 M β-mercaptoetanol), 66 μl of 1x ONPG (o-nitrophenyl-D-galactopyranoside, 4 mg/m) and 0.1 M sodium phosphate in total amount of 300 μl. The enzymatic reaction was stopped by the addition of 500 μl of 1 M Na_2_CO_3_. The relative β-galactosidase activity was determined by measuring absorbance of reaction mixtures at 420 nm and the obtained data were analyzed as described for the luciferase assays. The assays were performed as three independent experiments. Results are presented as means +/- standard deviations and two-tailed Student’s t test was used to determine P values (GraphPad InStat3 software).

### Immunoprecipitations

For co-immunoprecipitation of endogenous TFII-I with ectopically over-expressed Mdm2, HEK293T cells in 100-mm plates were transfected with the pCHDM1 plasmid coding for wild type Mdm2 using Lipofectamine 2000 reagent (Life Technologies) and treated 24 h post-transfection with proteasome inhibitor MG132 (Sigma-Aldrich, 15 μM in the complete DMEM medium supplemented with FCS and glutamine). 4 h later, cells were washed with ice-cold PBS and lysed on ice for 30 min in Triton X-100 lysis buffer (1% Triton X-100, 150 mM NaCl and 50 mM Tris pH 8.0) containing protease inhibitors (Complete Mini EDTA-free, Roche), and the extracts were cleared by centrifugation (13000 rpm/30 min/4°C). Immunoprecipitations were performed overnight at 4°C with 1 μg of anti-Mdm2 Ab-1 antibody (Merck-Millipore), followed by incubation with 20 μl of protein G-Sepharose beads (GE Healthcare) (45 min on a rotating wheel). Immunoprecipitated proteins were washed three times with the lysis buffer and the beads were resuspended in 2x SDS sample buffer. Proteins of interest in total cell lysates and immunoprecipitates were analyzed by SDS-PAGE followed by Western blotting.

For analyzing the same interaction on the level of endogenous proteins, eight confluent 100-mm dishes of H1299 cells were lysed as described above. Cleared cell lysates were divided and immunoprecipitations of endogenous Mdm2 and TFII-I were performed for 2 hours on a rotating wheel at 4°C with 2 μg of anti-Mdm2 antibody (HDM2-323, Santa Cruz Biotechnology) or 2 μg of mouse anti-TFII-I (BD Transduction Laboratories), respectively, together with 30 μl of protein G-Sepharose beads (GE Healthcare). Mouse α Tubulin antibody (Santa Cruz Biotechnology) was used as a negative control. Precipitated proteins were washed three times with the lysis buffer and the beads were resuspended in 2x SDS sample buffer. Proteins of interest in total cell lysates and immunoprecipitates were analyzed by SDS-PAGE followed by Western blotting.

### Immunofluorescence

U2OS cells grown on glass coverslips in 60-mm dishes were transfected with plasmids coding for Mdm2 and GST-TFII-IΔNLS using Lipofectamine 2000 (Life Technologies). 24 hours post-transfection, cells were treated for 3 h with 15 μM MG132 in DMEM supplemented with 10% FCS and glutamine, washed with PBS and fixed in 3% paraformaldehyde for 20 min at room temperature. After fixation, cell were washed with PBS, permeabilized with 0.2% Triton X-100 in PBS (5 min) and blocked with PBS containing 0.5% bovine serum albumin (30 min, room temperature). The coverslips were then incubated with primary antibodies recognizing Mdm2 (Ab-1, mouse monoclonal; Merck-Millipore) and the GST tag in TFII-I (Anti-GST antibody, goat polyclonal, GE Healthcare) for 2 h in the blocking solution. After three washes in PBS, coverslips were incubated for 1 h with a mixture of fluorescein isothiocyanate (FITC)-conjugated anti-goat and DyLight^TM^594 conjugated anti-mouse antibodies (Jackson ImmunoResearch Laboratories) in the blocking solution containing 1 μg/ml DAPI (Sigma-Aldrich) for visualization of cell nuclei. Coverslips were washed with PBS and mounted on microscopic slides using Vectashield mounting medium (Vector Laboratories). For the analysis of the effect of Mdm2 over-expression on subcellular localization of endogenous TFII-I, U2OS cells growing on glass coverslips were transfected with the Mdm2 expression plasmid pCHDM1A using Lipofectamine 2000, treated for 3 h with 15 μM MG132, and cover slips were processed as described above, with the exception that anti-TFII-I goat polyclonal antibody was used to detect endogenous TFII-I protein. Images were taken using FluoView^TM^ 500 confocal laser scanning fluorescence microscope (Olympus).

### SDS-PAGE and Western blot analysis

Total cell lysates and immunoprecipitates were resolved by SDS-polyacrylamide gel electrophoresis (SDS-PAGE) and blotted by a semi-dry transfer in an electric field onto polyvinylidene difluoride membranes (PVDF; Millipore). After transfer, the membranes were blocked in 5% low fat dry milk in TBST (10 mM TRIS-HCl, 100 nM NaCl and 0.05% Tween 20; pH 7.4) for 1 h at room temperature and incubated overnight at 4°C with a primary antibody in 1% low fat dry milk in TBST. The following primary antibodies were used: anti-TFII-I (V-18; Santa Cruz Biotechnology), anti-GST (GE Healthcare), anti-Mdm2 (Ab-1; Merck-Millipore), anti-Tubulin alpha (Exbio), anti-PCNA, (PC-10, kindly provided by Borivoj Vojtesek, Masaryk Memorial Cancer Institute, Brno, Czech Republic), anti-β-galactosidase (BG-02; Santa Cruz Biotechnology), anti-Myc (9E10, Santa Cruz Biotechnology), anti-Flag (M2; Sigma-Aldrich) and anti-GFP (Roche), using dilutions recommended by the manufacturers. After three washes in 1% milk in TBS, membranes were incubated one hour at room temperature with secondary antibodies conjugated to horseradish peroxidase (GE Healthcare). Enhanced chemiluminescence kit (ECL; GE Healthcare) and blue light sensitive medical X-ray film (Agfa) or G:BOX Chemi imaging system (Syngene) were used to visualize proteins of interest.

### Analyses of cell proliferation and viability

U2OS cells in 35-mm dishes were transfected with 1 μg of plasmid coding for enhanced GFP (pEGFP-C2) to mark the transfected cells, together with 3 μg of the β-galactosidase plasmid construct pcDNA4/LacZ or 3 μg of the TFII-I plasmid construct pcDNA4/TFII-I using Lipofectamine 3000 (Life Technologies). Cells were collected by trypsinization twenty-four hours post-transfection and both living and dead cells in each sample were pooled together and analyzed by flow cytometry using Attune Acoustic Focusing Cytometer (Life Technologies). The total count of GFP-positive cells and their proportion in the total cell population were determined, as well as the viability of cells using a standard viability assay based on propidium-iodide exclusion by intact viable cells. Untransfected U2OS cells grown under the same conditions served as a negative control.

## Results

### Mdm2 is a novel binding partner for TFII-I

**Fig 1:**
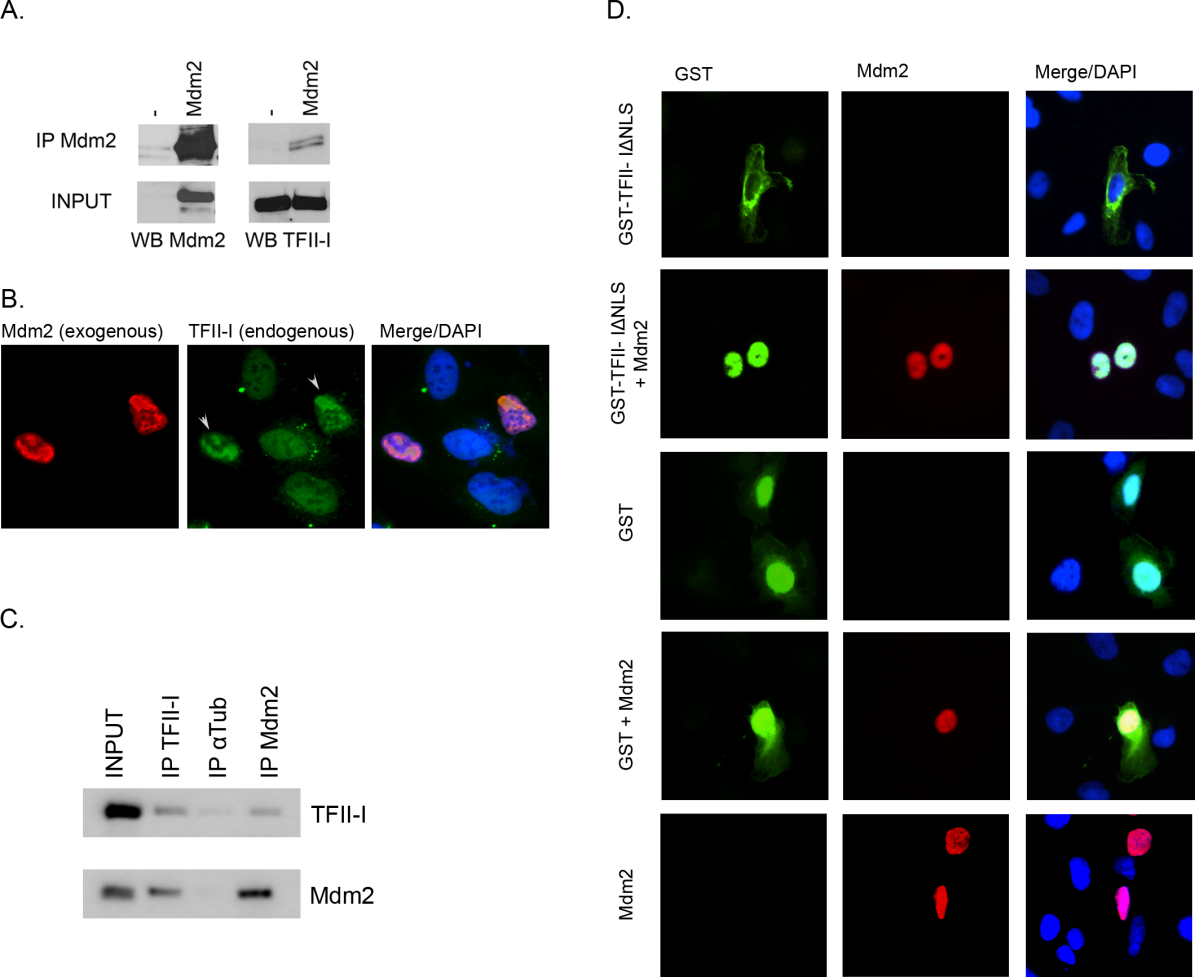
Mdm2 interacts with TFII-I. (A) HEK293T cells were transfected with a plasmid coding for human Mdm2. Endogenous TFII-I was co-immunoprecipitated from cell lysates using anti-Mdm2 antibody. (B) U2OS cells were transfected with a plasmid coding for human Mdm2. The subcellular localization of endogenous TFII-I and of the ectopically expressed Mdm2 was determined by immunofluorescence. DAPI was used to label cell nuclei. (C) Endogenous TFII-I and Mdm2 were co-immunoprecipitated from H1299 cell extracts using anti-TFII-I and anti-Mdm2 mouse monoclonal antibodies. Anti-tubulin α antibody served as a negative control. (D) Plasmids coding for GST and GST-tagged TFII-I mutant lacking the nuclear localization signal (ΔNLS) were transfected into U2OS cells either alone or together with Mdm2. Subcellular localization of the GST protein tag, GST-TFII-IΔNLS, and Mdm2 was analyzed using immunofluorescence. DAPI staining was used to label cell nuclei.

Several transcription factors and co-repressors involved in the regulation of cell growth and survival have been identified as binding partners for the Mdm2 oncogene, including p53, TAFII250, YY1, KAP1, E2F1, and HIF1α [27–33]. This known ability of Mdm2 to bind a number of transcription regulators, together with the report that TFII-I can be ubiquitinated in a p53-dependent manner in response to DNA damage [14], lead us to testing the possibility that the E3 ubiquitin ligase Mdm2 might physically interact also with the TFII-I transcription factor. As we had found that the endogenous levels of Mdm2 were relatively low in HEK293T cells, just at the detection limit of the anti-Mdm2 antibody used in our experiments, we transiently transfected the cells with a plasmid coding for human Mdm2. The anti-Mdm2 antibody was used to successfully co-immunoprecipitate the complex of Mdm2 with the endogenous TFII-I protein from lysates of transfected cells (Fig 1A).

In order to study the interaction between TFII-I and Mdm2 not just in cell lysates but also in intact in living cells, we transfected U2OS cells with the Mdm2 expression construct and determined the subcellular localization of the endogenous TFII-I by immunofluorescence. The endogenous TFII-I protein in the absence of ectopic Mdm2 was much more uniformly distributed throughout cell nuclei compared to cells expressing high levels of Mdm2. Ectopically over-expressed Mdm2 accumulated in distinct nuclear domains and endogenous TFII-I followed the pattern of Mdm2 localization in Mdm2-overexpressing cells (Fig 1B). These results suggested that Mdm2 and TFII-I might be able to interact also in intact cells.

To confirm the interaction between Mdm2 and TFII-I on the level of endogenous proteins, we performed immunoprecipitations with anti-Mdm2 and anti-TFII-I antibodies from a larger amount of cell extract, obtained by lysing eight confluent 100-mm dishes of H1299 cells. Results presented in Fig 1C indicate that a small amount of endogenous TFII-I was co-immunoprecipitated with endogenous Mdm2, and vice versa.

As yet another approach to show that TFII-I and Mdm2 might be able to interact not only in cell lysates but also in living cells, we designed a relocalization assay in which GST and a GST-tagged TFII-I mutant lacking the nuclear localization signal (TFII-IΔNLS) were ectopically expressed in human osteosarcoma U2OS cells, either alone or in combination with exogenous Mdm2. Unlike wild type TFII-I, which is predominantly nuclear, the majority of the TFII-IΔNLS mutant protein localized to the cytoplasm of transfected cells. Same as in the previous over-expression experiment, the Mdm2 protein localized predominantly to cell nuclei in U2OS cells. When TFII-IΔNLS was co-expressed together with Mdm2, we observed nuclear translocation of the TFII-I mutant in the majority of cells over-expressing Mdm2, suggesting that Mdm2 and TFII-I can indeed physically interact inside living human cells. In contrast, the GST protein subcellular distribution remained the same, both nuclear and cytoplasmic, in all transfected cells, regardless of the Mdm2 co-expression (Fig 1D).

### Mdm2 over-expression does not lead to TFII-I degradation

**Fig 2:**
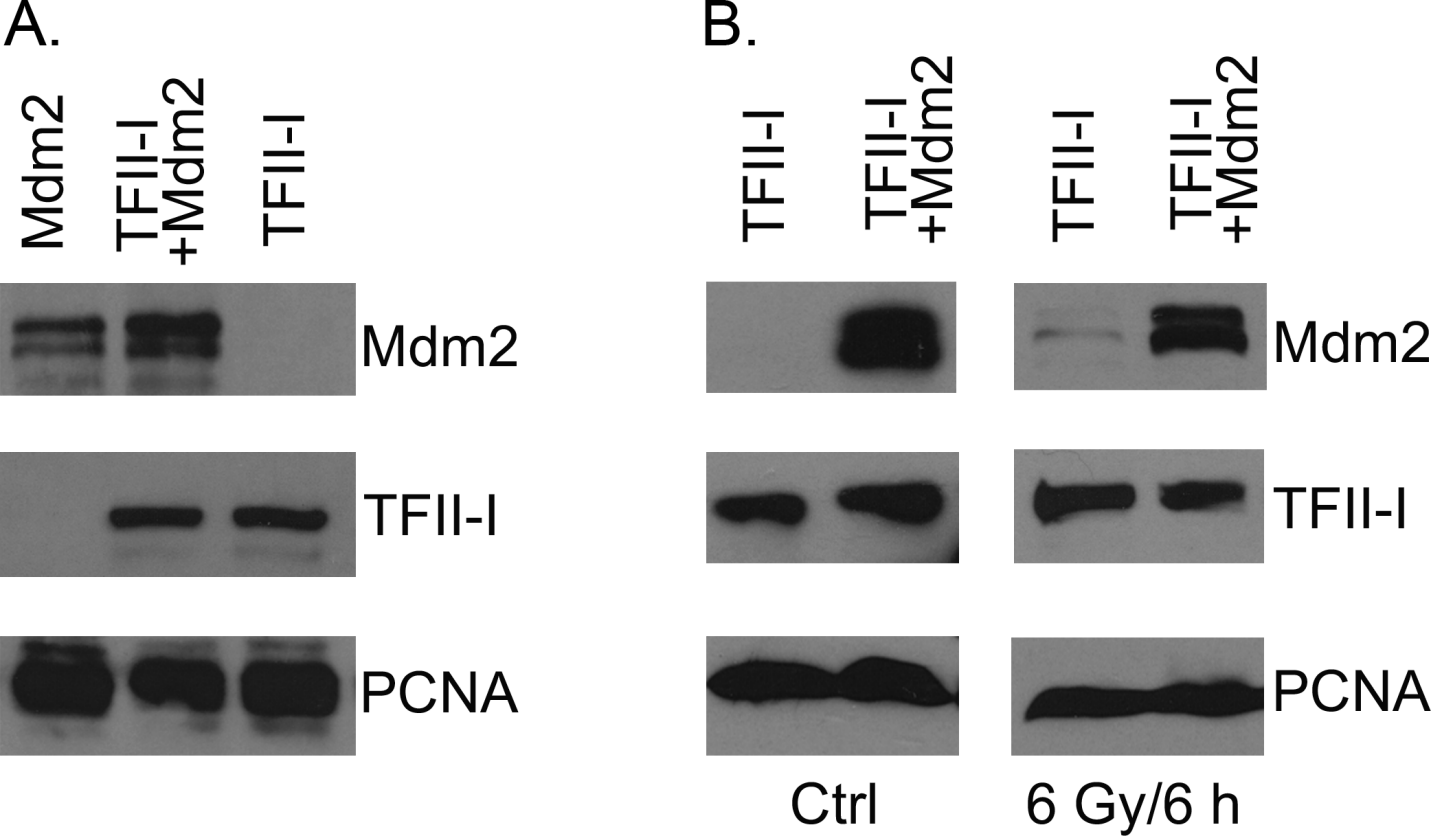
Mdm2 over-expression does not lead to TFII-I degradation. (A) U2OS cells were transfected with plasmids coding for TFII-I (pcDNA4/TFII-I) and Mdm2 (pCHDM1A) and changes in TFII-I protein levels 24 hours post-transfection were analyzed using SDS-PAGE and Western blotting. PCNA served as a loading control. (B) U2OS cells were transfected with plasmids encoding TFII-I (pcDNA4/TFII-I) and Mdm2 (pCHDM1A), irradiated with ionizing radiation (γ rays, 6Gy) 24 hours post-transfection, and lysed 6 hours later. Western blotting was used to compare TFII-I protein levels in cell lysates of unirradiated controls and irradiated samples, both in the absence and in the presence of Mdm2 over-expression.

The expression of E3 ubiquitin ligase Mdm2 is regulated by the transcription activity of tumor suppressor p53. In order to determine whether Mdm2 might play a role in the previously reported p53-dependent TFII-I degradation in response to DNA damage [14], we transfected U2OS cells with a TFII-I expression plasmid, alone or in combination with the excess of plasmid encoding Mdm2. As we did not detect any significant change in TFII-I protein levels in cells over-expressing exogenous Mdm2 (Fig 2A), we also tested the possibility that Mdm2 could regulate TFII-I levels specifically in response to DNA damage. Also in this experiment we did not detect any decrease of TFII-I protein levels when cells expressing ectopically TFII-I and Mdm2 were treated with ionizing radiation (Fig 2B).

### TFII-I regulates CMV promoter activity

**Fig 3:**
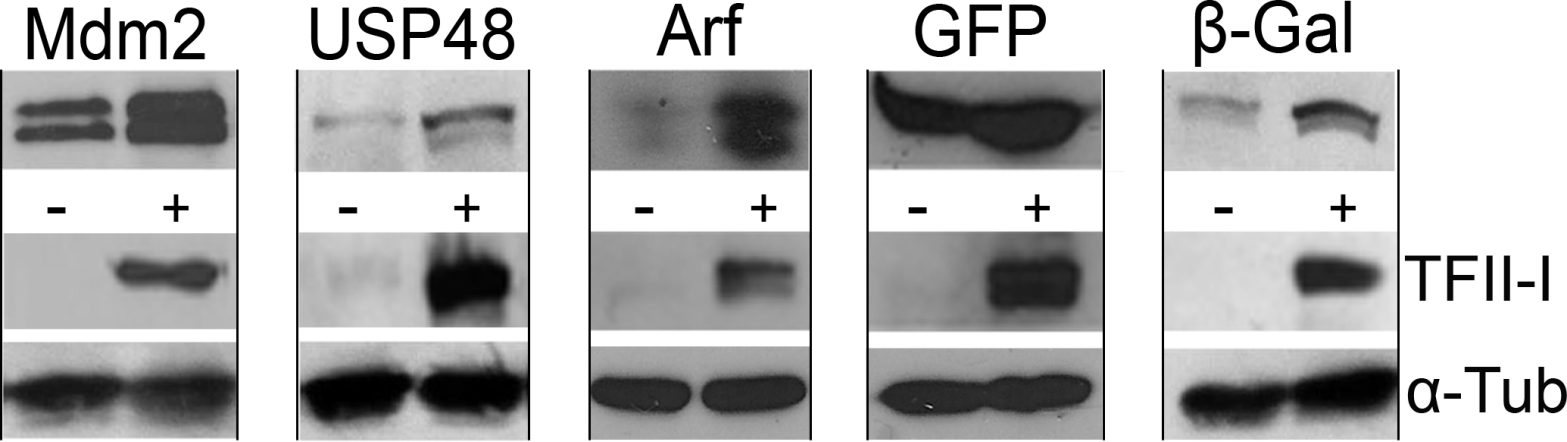
TFII-I enhances gene expression from plasmid constructs containing CMV promoter. Plasmid constructs coding for human oncogene Mdm2, human deubiquitinase USP48, human tumor suppressor p14 (Arf), jellyfish green fluorescent protein (GFP), and bacterial β-galactosidase (β-Gal) under the control of the CMV promoter were transfected into U2OS cells alone or in combination with pcDNA4/TFII-I. Whole cell extracts were resolved by SDS-PAGE and protein expression was analysed by Western blotting. Alpha-tubulin (α-Tub) served as a loading control.

During our experiments we observed an unexpected increase of Mdm2 protein levels in the presence of ectopically expressed TFII-I (Fig 2A), possibly indicating that TFII-I protein, when over-expressed, might interfere with Mdm2 auto-ubiquitination and/or its degradation in 26S proteasomes. However, the subsequent experiments showed that TFII-I co-transfection induced increase in the levels of other ectopically expressed proteins that were unrelated to Mdm2 and as diverse as the human deubiquitinating enzyme USP48, human tumor suppressor p14 (Arf), jellyfish green fluorescent protein (GFP), and bacterial β-galactosidase (LacZ), suggesting that the observed effect was more general, not specific to any particular protein (Fig 3).

**Fig 4:**
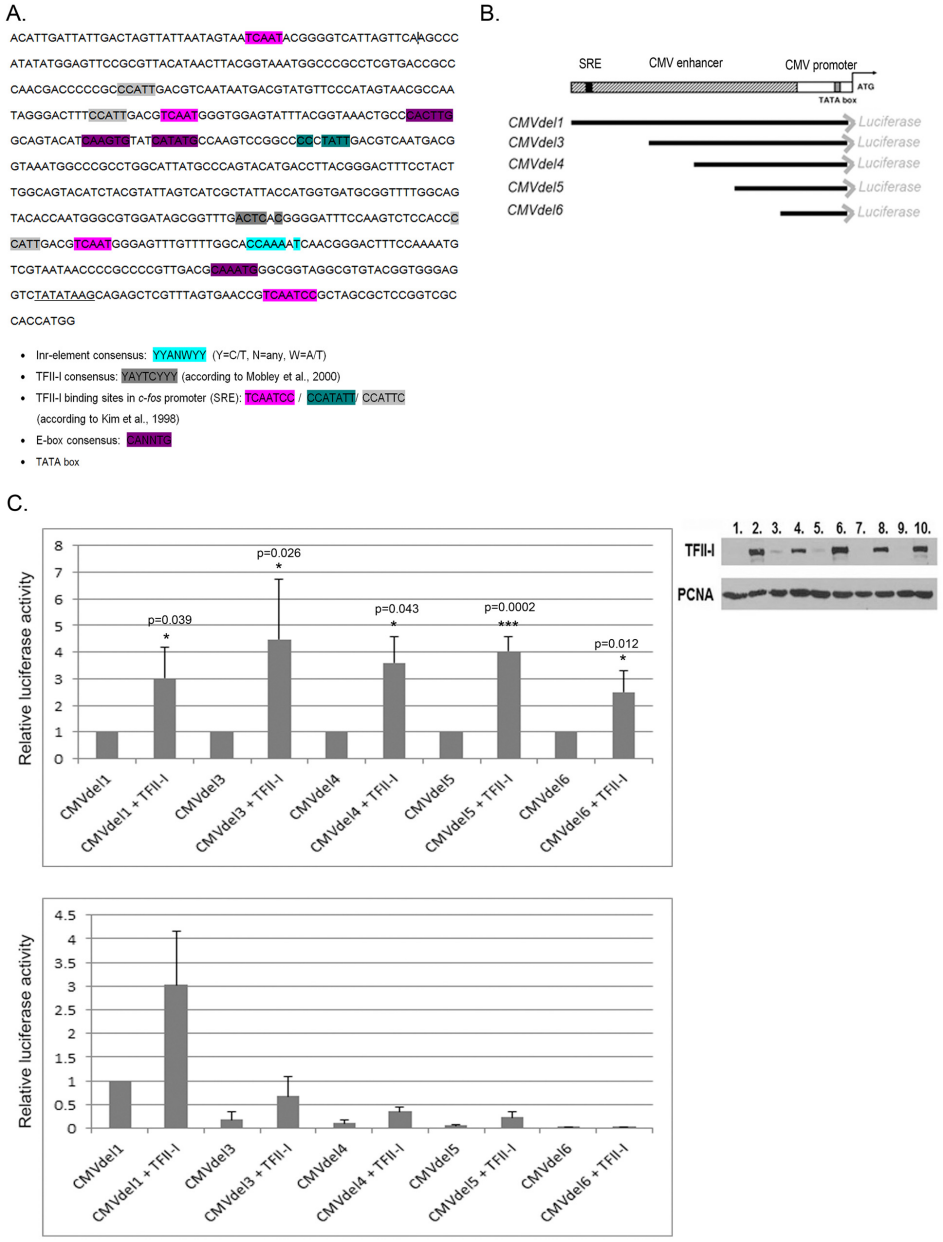
Ectopic expression of TFII-I activates CMV promoter. (A) CMV promoter sequence with potential TFII-I binding sites. (B) Schematic representation of CMVdel luciferase vectors used in this study. (C) CMVdel series vectors were transfected into U2OS cells, either alone or in combination with pcDNA4/TFII-I and luciferase activity was measured in cell extracts 24 h post-transfection. The upper graph shows the relative changes in luciferase activity induced by TFII-I for each construct of the series individually. Data obtained in three independent experiments are presented as mean +/- standard deviation (* P<0.05, *** P<0.001). The obtained P values suggest significant differences in luciferase activity between cells co-transfected with TFII-I and cells transfected only with the respective luciferase construct. The lower graph shows the same set of results presented in relation to the full-length construct (activity in cells transfected with CMVdel1 was set as 1). Representative Western blot shows TFII-I protein levels in each sample (loaded in the same order as in the graphs). Endogenous PCNA served as a loading control.

The only apparent link between the different proteins up-regulated in response to TFII-I over-expression was the fact that they were expressed from plasmid constructs containing the immediate-early promoter/enhancer region of human cytomegalovirus (the so called CMV promoter), a regulatory DNA element that is widely used in plasmid and viral constructs in basic biomedical research as well as in experimental gene therapy. By analyzing the CMV promoter sequence we identified several potential TFII-I binding sites corresponding to, or closely related to the known TFII-I binding sequences (Fig 4A). In order to confirm results indicating that TFII-I could serve as a novel regulator of the CMV promoter, we used a set of plasmid constructs in which the expression of luciferase reporter gene was under the control of the full-length CMV promoter or its shorter deletion mutants [23] (Fig 4B).

U2OS osteosarcoma cells were transfected with the individual CMV promoter reporter constructs, either alone or in combination with a TFII-I expression plasmid, and the activity of luciferase was measured in lysates of transfected cells. Results presented in Fig 4C show that the relative transcriptional activity of each individually analyzed promoter construct was up-regulated when TFII-I was co-expressed, indicating the presence of several binding sites within the CMV promoter sequence that can respond to TFII-I protein. However, the comparison of the relative activity between the different constructs showed that the full-length promoter construct (CMVdel1) induced significantly higher levels of luciferase expression than constructs containing shorter versions of the CMV promoter, both on its own and when exogenous TFII-I was co-expressed. These results confirm that ectopically expressed TFII-I is a novel activator of the CMV promoter and that 5’ portion of the CMV promoter is indispensable for its maximal activity, both in the absence and in the presence of ectopically expressed TFII-I.

**Fig 5:**
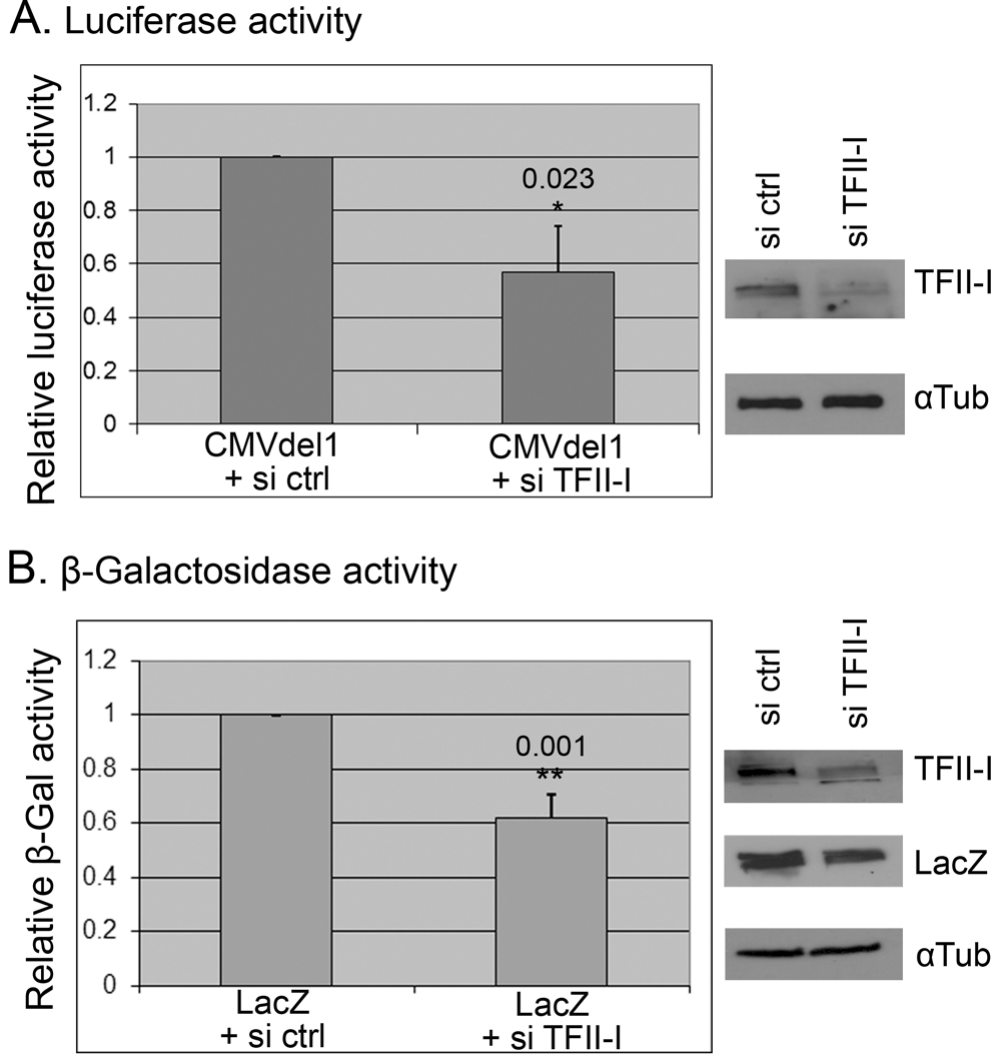
Endogenous TFII-I contributes to CMV promoter activity in human cells. U2OS cells were transfected with a mixture of siRNAs targeting TFII-I (si TFII-I) or non-targeting control siRNAs (si ctrl). Twenty four hours later, the same cells were transfected again, this time with the CMVdel1 luciferase construct (A) or the β-galactosidase construct pcDNA3.1(-)/myc-His/LacZ (LacZ) (B). Cells were lysed 24 hours later. The graphs show the relative changes in luciferase activity or β-galactosidase activity, respectively, in three independent experiments (mean +/- standard deviation; * P<0.05, ** P<0.01). The representative results of Western blotting analysis illustrate the efficiency of TFII-I knock down in the presented experiments.

In the next experiment we performed siRNA-mediated knock-down of TFII-I expression in order to test the possibility that normal cellular levels of TFII-I might contribute to CMV promoter activity in human cells. We compared luciferase expression from the full length luciferase plasmid (CMVdel1) in U2OS cells transfected with control non-targeting siRNAs and in cells transfected with a mixture of TFII-I-specific siRNAs, and we observed a significant drop of luciferase activity in cells with decreased TFII-I protein levels (Fig 5A). Similarly, β-galactosidase activity in the lysates of U2OS cells transfected with pcDNA3.1(-)/myc-His/LacZ plasmid was significantly lower in TFII-I siRNA–transfected cells compared to cells transfected with a mixture of control non-targeting siRNAs (Fig 5B). Similar results, strongly suggesting that endogenous TFII-I protein contributes to CMV promoter activity in human cells, were obtained in three independent experiments.

### Mdm2 inhibits TFII-I-directed transcription

**Fig 6:**
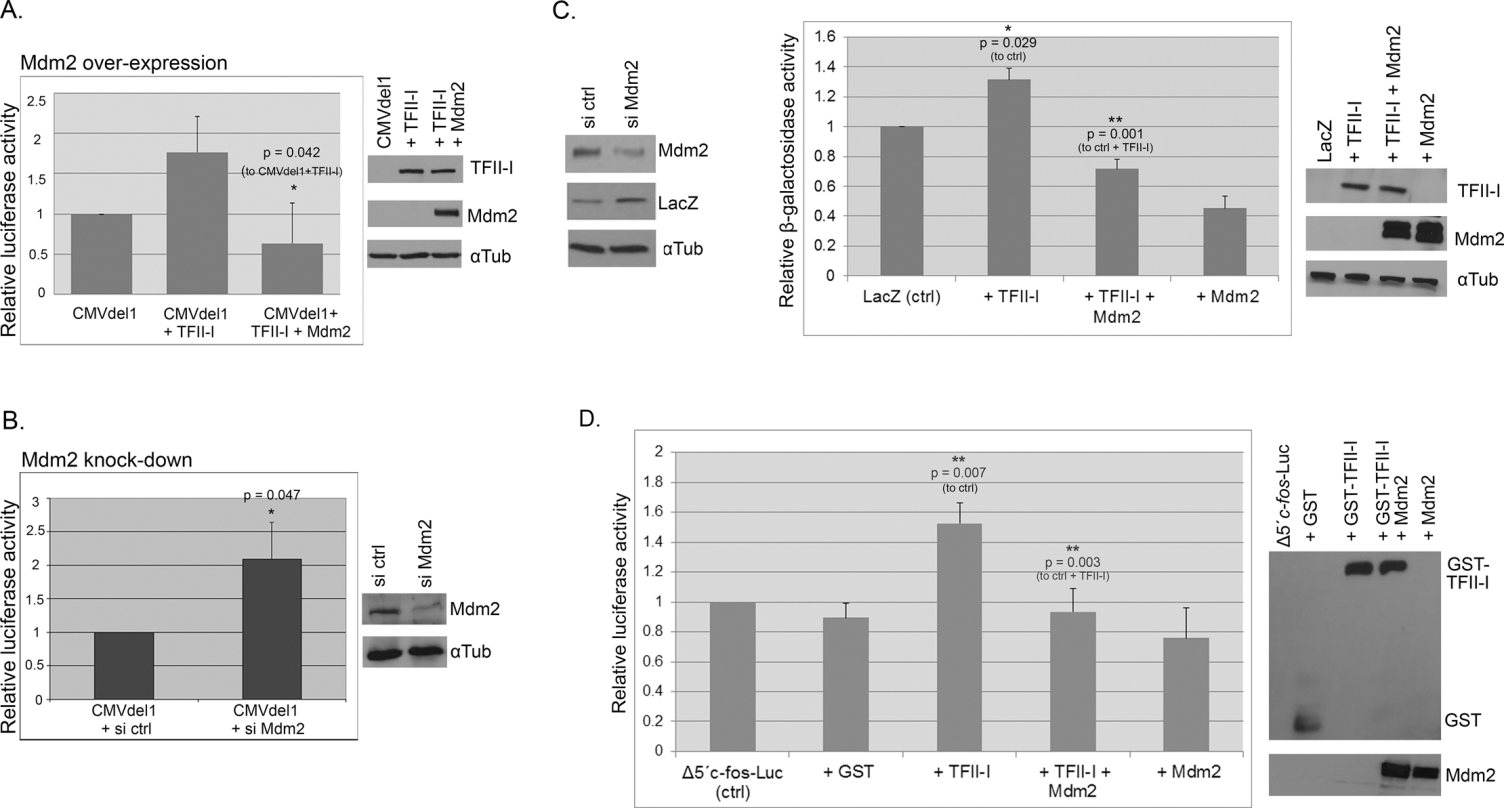
Mdm2 regulates TFII-I-mediated transcription in a p53-independent manner. (A) Mdm2 inhibits TFII-I–mediated upregulation of transcription from the CMV promoter. U2OS cells were transfected with the CMVdel1 luciferase plasmid either alone or together with plasmids coding for TFII-I (pcDNA4/TFII-I) and Mdm2 (pcHDM1A). Luciferase activity was measured 24 h post-transfection. (B) Endogenous Mdm2 levels modulate CMV promoter activity. The luciferase plasmid CMVdel1 was transfected into U2OS cells, twenty four hours after the cells had been transfected with either non-targeting control siRNAs or siRNAs targeting Mdm2. Luciferase activity in cell lysates was measured twenty four hours later. Both graphs show results from three independent experiments (mean +/- standard deviation; * P<0.05). (C) TFII-I and Mdm2 do not require p53 to regulate CMV promoter. H1299 cells (p53 null) were transfected with the β-galactosidase construct pcDNA3.1(-)/myc-His/LacZ, twenty four hours after the cells had been transfected either with non-targeting control siRNAs or siRNAs targeting Mdm2. Cells were lysed 24 hours later and β-galactosidase expression (LacZ) was analyzed by Western blotting (left panel). To determine the effect of TFII-I on CMV promoter in p53-null cells more precisely, H1299 cells were transfected with with pcDNA3.1(-)/myc-His/LacZ alone, with pcDNA4/TFII-I, or together with plasmids encoding both TFII-I and Mdm2 (pcHDM1A). The activity of β-galactosidase was measured 24 h later (right panel). Data obtained in three independent experiments are presented as mean +/- standard deviation (* P<0.05, ** P<0.01). (D) Mdm2 can influence the expression of cellular TFII-I targets. GST-TFII-I was expressed from pEBG/TFII-I in the presence or absence of ectopic Mdm2 expression in U2OS cells, along with the TFII-I-responsive *c-fos* promoter luciferase construct. The graph presents *c-fos* promoter transcription activity obtained in luciferase assays in three independent experiments (mean +/- standard deviation; ** P<0.01). The empty vector pEBG (GST) was used as a negative control.

While we did not observe any direct effect of Mdm2 on TFII-I levels, there still was the possibility that the interaction between Mdm2 and TFII-I might influence TFII-I–driven transcription. To test it, we expressed wild type TFII-I together with Mdm2 and the CMVdel1 construct and measured luciferase activity in cell lysates twenty-four hours post-transfection. Results presented in Fig 6A show that Mdm2 over-expression had inhibitory effect on TFII-I–driven transcriptional activation of the CMV promoter. To find out whether the relatively low endogenous levels of Mdm2 could also have influence on CMV promoter activity, we compared luciferase activity in U2OS cells transfected with the CMVdel1 luciferase plasmid twenty-four hours after the cells had been transfected with either non-targeting control siRNAs or siRNAs targeting Mdm2 expression. Results presented in Fig 6B indicate that changes in endogenous Mdm2 protein levels can have a significant impact on the CMV promoter activity in human cells.

As Mdm2 is best known as the major regulator of the stability and transcriptional activity of p53 tumor suppressor that has numerous target genes and is involved in the regulation of many different cellular functions. Therefore, it was important to test the validity of our findings in a p53-deficient background. We started by testing whether endogenous Mdm2 can regulate CMV promoter in the absence of p53. In p53-null H1299 lung cancer cells transfected with pcDNA3.1(-)/myc-His/LacZ plasmid, Mdm2 knock-down increased β-galactosidase protein levels (Fig 6C, left). Using the same cell line and the β-galactosidase expression plasmid, we analyzed the effect of TFII-I and Mdm2 over-expression on CMV promoter activity in the p53-negative cellular context more quantitatively. Data presented in Fig 6C (right) suggest that neither the ability of TFII-I to transcriptionally activate the CMV promoter nor the negative effect of Mdm2 on this activation requires the presence of p53.

In the above-described experiments we concentrated primarily on the CMV promoter, to characterize it as a new TFII-I target. However, having shown that Mdm2 was able to interfere with TFII-I transcriptional activity independently of p53, we were interested in finding out whether the expression of normal cellular targets of TFII-I might be influenced by the levels of Mdm2. We examined the effect of Mdm2 on the TFII-I-mediated transcriptional regulation of the c-*fos* promoter using pSVOAΔ5’*c-fos*-Luc plasmid, containing a 379 bp fragment of murine *c-fos* promoter upstream of the luciferase reporter gene, that was used to determine TFII-I transcriptional activity in other studies [11,34,35]. U2OS cells were transfected with the pSVOAΔ5’*c-fos*-Luc reporter plasmid and GST-TFII-I alone or in combination with Mdm2. As expected, TFII-I stimulated the transcriptional activity of the *c-fos* promoter. Upon co-expression of Mdm2, the TFII-I-mediated increase *c-fos* promoter transcription was lost, indicating that Mdm2 can have negative effect not only on the TFII-I-mediated transcription of viral promoters but also on endogenous TFII-I target genes (Fig 6D).

**Fig 7:**
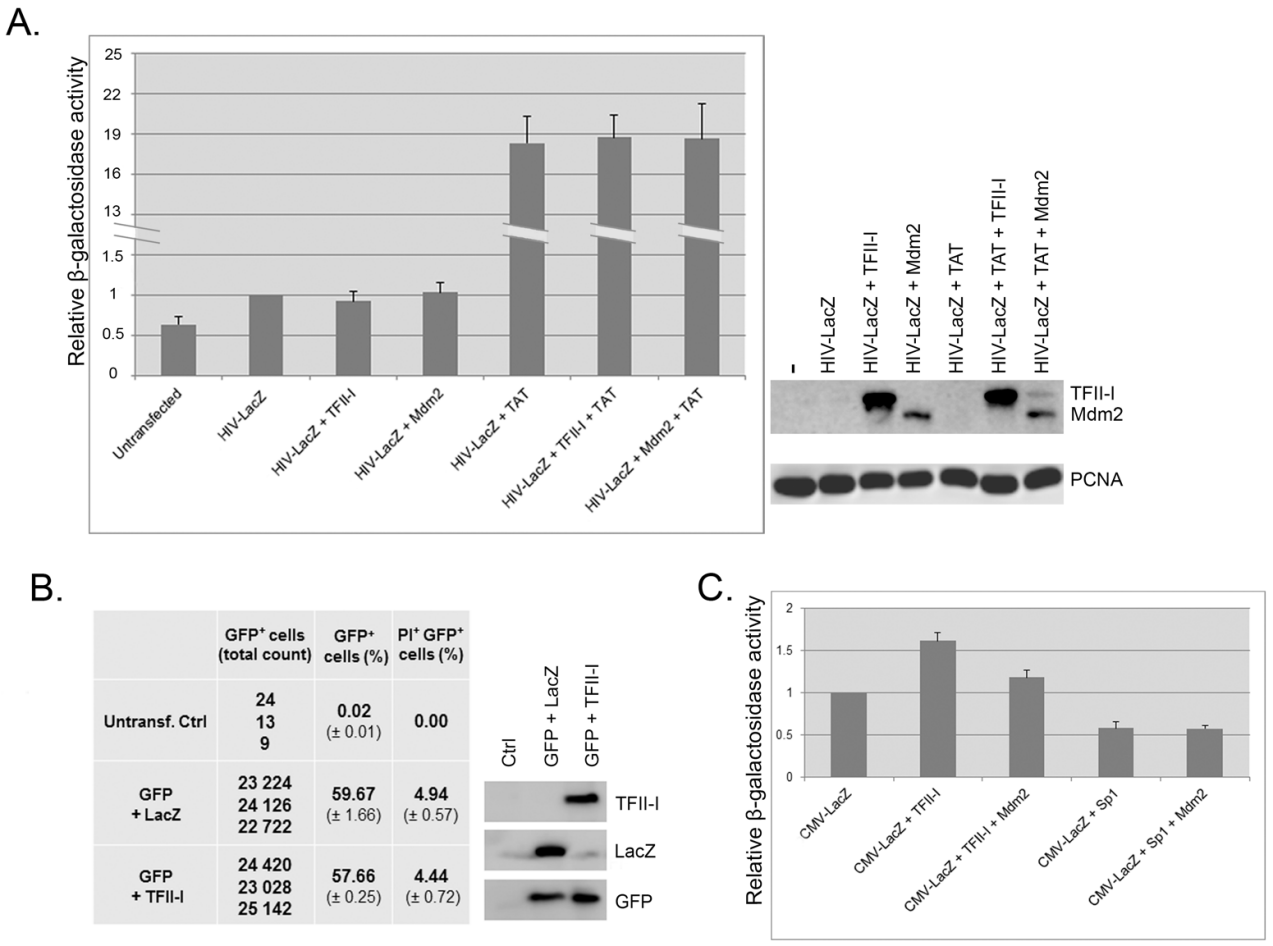
Effects of TFII-I and Mdm2 on transcription are specific to CMV promoter. (A) Over-expression of TFII-I and Mdm2 does not influence the activity of HIV 3’ LTR promoter. H1299 cells were transfected with pHIVlacZ (HIV-LacZ) alone or with HIV-LacZ plus pCEP4-Tat (TAT), together with plasmids coding for TFII-I (pcDNA4/TFII-I) and Mdm2 (pcHDM1A). The activity of β-galactosidase was measured 24 h later. Data obtained in three independent experiments are presented as mean +/- standard deviation. Representative Western blot shows TFII-I and Mdm2 protein levels in lysates of transfected cells. Endogenous PCNA served as a loading control. (B) Over-expression of TFII-I does not induce significant changes in cell proliferation or viability. U2OS cells were transfected with pEGFP-C2 to mark the transfected cells with GFP, together with either pcDNA4/LacZ (GFP+LacZ) or with pcDNA4/TFII-I (GFP+TFII-I). Flow cytometry was used 24 h post-transfection to determine the proportion of GFP-positive cells in the total cell population and cell viability in a PI exclusion assay. The experiment was performed in triplicates and the table shows the real counts of GFP-positive cells, their proportion in total cell population (mean) and the proportion of dead transfected cells (PI-positive, GFP-positive) in total cell population (mean). Untransfected U2OS cells served as a negative control. The Western blot shows GFP, TFII-I and Mdm2 expression in total cell lysates of transfected cells. (C) Sp1 does not require Mdm2 co-expression for inhibiting CMV promoter. H1299 cells were transfected with pcDNA4/LacZ alone, with pcDNA4/LacZ plus pcDNA4/TFII-I or pcDNA4/LacZ plus pEGFP-Sp1, together with plasmid coding for Mdm2 (pcHDM1A). The activity of β-galactosidase was measured 24 h later (right panel). Data obtained in three independent experiments are presented as mean +/- standard deviation

While the above-presented data suggested a role for TFII-I and Mdm2 in CMV promoter regulation, it was important to exclude the possibility that the observed effects were the result of global changes in gene expression, caused by TFII-I or Mdm2 interactions with cellular transcription machinery. We transfected H1299 cells with pHIVLacZ plasmid construct for HIV promoter-driven β-galactosidase expression either alone or together with HIV trans-activator of transcription (Tat), and co-expressed TFII-I or Mdm2. The HIV promoter not only did not respond to TFII-I but also was not influenced by increased Mdm2 levels (Fig 7A), suggesting that the effects of TFII-I and Mdm2 on the CMV promoter were specific. The same experiment was performed also in HEK293 cells and again it did not show any significant changes in HIV promoter activity in the presence of TFII-I or Mdm2 (not shown).

In addition, we performed an experiment designed to confirm that the above-presented results were not caused by unspecific effects of TFII-I overexpression on cell proliferation or viability. U2OS cells were transfected either with pcDNA4/LacZ (control) or with pcDNA4/TFII-I. Plasmid construct pEGFP-C2 was included in the transfection mixtures to mark the transfected cells with GFP and untransfected U2OS cells served as a negative control. Twenty-four hours post-transfection we used flow cytometry to determine the proportion of GFP-positive cells in cell population and cell viability in a PI exclusion assay. Results presented in Fig 7B clearly show that TFII-I over-expression did not have a significant effect on U2OS proliferation or cell viability. Similar results showing no effect on cell proliferation or viability were obtained when the experiment was performed in H1299 cells (not shown).

In the next experiment, we compared the effects of TFII-I and Sp1 transcription factors on the activity of CMV promoter. The reason for that were previous publications showing the presence of Sp1 consensus binding sites in the CMV promoter [36] and the ability of Mdm2 to physically interact with Sp1 and inhibit Sp1-mediated transcription [37]. Therefore, Mdm2 interaction with Sp1 and inhibition of its activity could be partly responsible for the observed effect of Mdm2 on CMV promoter activity. However, results presented in Fig 7C show that ectopic expression of Sp1 had a strong negative impact on CMV promoter activity in H1299 cells and Mdm2 co-expression was not required for CMV inhibition by Sp1. Similar results were obtained in HEK 293 cells (not shown). These data indicate that Sp1 might act as a negative regulator of the immediate/early CMV promoter constructs independently of its physical interaction with Mdm2. This might indicate that the observed negative effect of Mdm2 on TFII-I-induced CMV promoter activity was not mediated by Mdm2-Sp1 interaction inhibiting the Sp1 transcriptional activity.

## Discussion

The major immediate-early enhancer/promoter of human cytomegalovirus is one of the most robust transcriptional control elements for the ectopic expression of transgenes in mammalian cells and is used in a variety of viral and plasmid DNA vectors [38]. Since the CMV promoter can drive high constitutive expression levels in a wide range of human tissues, it is used in a large proportion of DNA vaccines (reviewed in [39] and represents an important tool for experimental gene therapy, despite considerable evidence that viral promoters are prone to inactivation and silencing in mammalian cells *in vitro* and *in vivo* (reviewed in [40]).

The CMV promoter contains known binding sites for several transcription factors, including NF-κB/rel, CREB/ATF, Sp1, AP1, retinoic acid receptor, ETS and SRE [41]. In the current study, we present data suggesting that the Williams-Beuren syndrome–associated transcription factor TFII-I is an important regulator of gene expression driven by the CMV promoter in human cells. Co-transfection of TFII-I strongly enhanced expression of several unrelated genes from plasmid constructs containing the CMV promoter. Sequence analysis and subsequent use of a series of luciferase expression constructs with deletions in the CMV immediate early promoter/enhancer indicated the presence of several TFII-I binding motifs within the CMV promoter.

While the loss of the 5’ portion of the promoter/enhancer sequence (CMVdel3–6) had a clear impact on the transcriptional activity and on the TFII-I response in absolute terms, a strong relative increase of the basal activity was observed also in constructs CMVdel4 and CMVdel5 that retained only the 3’ half of the CMV promoter sequence. This suggests that the 3’ region of CMV promoter contains functional TFII-I-binding sequences as well. Importantly, not only the ectopic over-expression of TFII-I but also the siRNA-mediated knock-down of the endogenous TFII-I expression had a significant impact on the CMV promoter activity in our experiments, suggesting that normal cellular levels of TFII-I participate in the regulation of CMV promoter in human cells.

Mdm2 serves as an E3 ubiquitin ligase for tumor suppressor p53 and is critically important for keeping p53 protein levels and activity low under non-stressed conditions. At the same time, Mdm2 expression in normal cells is regulated by p53, thereby creating an auto-regulatory feedback loop that is often disrupted in cancer cells by Mdm2 over-expression, leading to the loss of p53 tumor-suppressive function (for review see [16]). In addition to p53, Mdm2 protein has been reported to interact with several other transcription regulators, including TAFII250, YY1, KAP1, E2F1, and HIF1α [27–33]. Our results show that Mdm2 can bind to TFII-I and inhibit TFII-I–mediated activation of transcription, representing a novel p53-independent role for Mdm2. In human cancers with *Mdm2* gene amplification the negative effect of Mdm2 on TFII-driven transcription could disrupt normal cellular functions of TFII-I. At the same time, it could have a negative impact on the success of experimental gene therapy using CMV promoter-derived vectors.

Interestingly, the p53 tumor suppressor has recently been reported as a direct negative regulator of CMV promoter activity in mammalian cells [42]. Taking into account our findings, it seems possible that, despite having opposing roles in the regulation of cell growth and survival, both p53 and Mdm2 could contribute to the negative control of CMV promoter activity in normal, untransformed cells. On the other hand, Mdm2 over-expression in cancer cells negatively regulates p53 levels and increased Mdm2 levels would probably abrogate any inhibitory effect of p53 on the CMV promoter activity. Moreover, the observed changes in CMV-driven β-galactosidase expression in p53-null H1299 cells in response to TFII-I over-expression and Mdm2 knock-down speak against any requirement for direct involvement of p53 in the mechanisms described in this paper.

We cannot fully exclude the possibility that other cellular transcription factors known to interact with Mdm2, such as YY1, E2F1, TAFII250 or Sp1 might contribute to the observed inhibitory effect of Mdm2 on CMV promoter activity independently of p53 and TFII-I. For example, Sp1 consensus binding sites were identified in the CMV promoter [36] and Mdm2 protein was shown to physically interact with Sp1 and inhibit Sp1-mediated transcription [37]. The possible involvement of Mdm2 in CMV promoter regulation could be complicated even more by the reported ability of Mdm2 to activate gene expression through directly binding the Sp1 binding sites in gene promoters [17]. Nevertheless, our observation that most if not all of the increase of CMV promoter activity induced by TFII-I is eliminated when Mdm2 is co-expressed could suggest that TFII-I inhibition contributes significantly to Mdm2 effects on CMV promoter activity. Moreover, when we tested the effect of Sp1 on CMV promoter we found that GFP-Sp1 expression does not activate the CMV promoter but inhibits its activity even in the absence of Mdm2 co-expression, suggesting that this effect of Sp1 is Mdm2 independent.

While the results of our immunoprecipitations and relocalization assays suggest that Mdm2 and TFII-I could physically interact or at least co-localize within the same protein complex, exactly how Mdm2 inhibits TFII-I activity in the absence of any apparent TFII-I degradation remains to be elucidated. A possible explanation could offer the mechanism by which Mdm2 regulates its most-studied target–the tumor suppressor p53. In addition to mediating p53 ubiquitination and targeting p53 for proteasomal degradation, a physical interaction between the N-terminus of Mdm2 and the N-terminal transactivation domain of p53 efficiently inhibits p53-mediated transcription [43,44]. A similar strategy is adopted also by the closely related oncoprotein MdmX. Even though it lacks the E3 ubiquitin ligase activity, its overexpression often leads to inactivation of p53 tumor suppressor in human cancers by binding to the p53 transactivation domain and inhibiting p53-directed transcription [45,46]. Moreover, the binding of the central acidic domain of Mdm2 to p53 can induce a change of p53 conformation that prevents the binding of p53 to target DNA sequences [47]. Another possible explanation for the decrease of TFII-I-mediated transcription in the presence of Mdm2 could be a global negative effect of Mdm2 over-expression on gene transcription in general. However, in the light of our data, this does not seem to be very probable as HIV-driven β-galactosidase expression not only did not respond to TFII-I, but also was not influenced by increased Mdm2 levels.

Recent evidence suggests that human cytomegalovirus might play a role in modulating tumor microenvironment as well as in the initiation of tumor cells themselves. In the case of glioblastoma multiforme, a highly malignant primary central nervous system neoplasm characterized by tumor cell invasion, robust angiogenesis, and a mean survival of only 15 months, about 90 per cent of the cases are positive for HCMV [48–50]. While the exact role of HCMV infection in glioblastoma pathogenesis remains unclear, pp71, a cytomegaloviral protein previously shown to promote cell cycle progression, is present in the majority of human glioblastomas and is preferentially expressed in the CD133+ cancer stem-like cell population [51]. The *Mdm2* gene was found amplified or over-expressed in about 10 per cent of glioblastomas [52,53]. In the light of our findings, it could be of interest to analyze the patterns of Mdm2 expression and HCMV positivity in glioblastomas and establish whether strong Mdm2 expression might correlate with the absence of HCMV infection or the expression of viral proteins. However, HCMV might not be the only virus that could be regulated by the interplay between TFII-I and Mdm2. There are reports that TFII-I is involved in the regulation of the long terminal repeat (LTR) promoters of retroviruses, specifically the Rous sarcoma virus [54] and the human immunodeficiency virus (HIV-1) [55–57]. More recent studies have shown that TFII-I–mediated transcription is required for HIV-1 virus production and TFII-I silencing leads to decreased HIV production in T cells [58,59]. In this study, we tested the responses to TFII-I and Mdm2 of a portion of the 3’ LTR promoter of HIV-1. The HIV promoter was active in human cancer cells but did not respond to either TFII-I or Mdm2. Whether the functional interaction between Mdm2 and TFII-I reported in this paper might have any significant effect on the latency, transcription or virion production of HIV and other retroviruses still remains to be determined. Interestingly, cellular TFII-I has only recently been identified as a negative regulator of the L4 promoter of human adenovirus type 5, widening the spectrum of viral TFII-I targets and suggesting that TFII-I might play different roles in the regulation of gene expression in different viruses [60].

Taken together, our work suggests that oncogene Mdm2 is a new binding partner for the multifunctional transcription factor TFII-I. A functional interplay between these two proteins can regulate the activity of both cellular and viral TFII-I targets, including the widely used CMV promoter, which we newly identified as a transcriptional target of TFII-I in human cells.
